# Social and Medical Risk Factors Associated with Supportive Needs in the First Year Following Localized Prostate Cancer Treatment

**DOI:** 10.1007/s11764-020-00916-5

**Published:** 2020-07-18

**Authors:** Erin K. Tagai, Shawna V. Hudson, Michael A. Diefenbach, Jenny Xu, Alicja Bator, Allison Marziliano, Suzanne M. Miller

**Affiliations:** 1 Fox Chase Cancer Center, 333 Cottman Ave, Philadelphia, PA, 19111; 2 Rutgers Robert Wood Johnson Medical School, Rutgers, the State University of New Jersey, 125 Patterson St, New Brunswick, NJ, 08901; 3 Division of Population Science, Rutgers Cancer Institute of New Jersey, Rutgers, the State University of New Jersey, 195 Little Albany St, New Brunswick, NJ, 08903; 4 Center for Health Innovation and Outcomes Research, Feinstein Institute for Medical Research, Northwell Health, 300 Community Dr, Manhasset, NY, 11030

**Keywords:** cancer survivorship, coping, prostate cancer, social determinants of health, self-efficacy

## Abstract

**Purpose:**

Individuals who completed treatment for prostate cancer (PCa) often report poor coping and practical concerns when adapting to new roles in their lives—and strong patient-provider communication is critical for this period. However, there is limited research identifying factors associated with supportive needs after the completion of PCa treatment. This study aimed to identify the social and medical risk factors associated with supportive needs for adapting among individuals who completed treatment for localized PCa.

**Methods:**

Using baseline data from a study evaluating a web-based support system for patients in the first year following treatment for localized PCa, self-efficacy for re-entry (e.g., maintaining relationships, symptom management), medical interactions, and practical concerns (e.g., insurance, exercise) were assessed. Multivariable regression analyses were completed to identify risk factors for low readiness.

**Results:**

Participants (N=431) with lower health literacy or income, or with depressive symptoms had lower self-efficacy for re-entry, more negative interactions with medical providers, and more practical concerns (*ps*<.05). Lastly, Non-Hispanic White participants reported greater readiness compared to all other races (*ps*<.05).

**Conclusions:**

Multiple social and medical risk factors are associated with greater supportive needs when adapting to new roles after PCa treatment. Understanding the risk factors for supportive needs in this period is critical. Future research is needed to help providers identify and support individuals at risk for poorer coping and greater practical concerns after treatment completion.

**Implications for Cancer Survivors:**

Identifying individuals with greater supportive needs following treatment for localized PCa treatment will help ensure successful adaptation to new roles.

Prostate cancer (PCa) is the most common cancer among men in the United States with 90% of PCa diagnoses found at a local or regional stage [1]. Localized PCa has a 5-year relative survival rate nearing 100%, resulting in a growing number of healthy individuals with a history of PCa [2]. Individuals with localized PCa typically have the choice between active treatment (e.g., surgery, radiation therapy) and active surveillance [3]. Despite the growing number of localized PCa patients choosing active surveillance, most localized PCa patients in the U.S. undergo active treatment, [3] often resulting in urinary, bowel, and sexual dysfunction side effects [4, 5]. PCa patients that underwent surgery most commonly report post-treatment urinary and sexual dysfunction, while those that underwent radiation therapy most commonly report short-term bowel dysfunction [4]. Despite functional side effects often diminishing over time, some individuals experience persistent side effects requiring surgical intervention [4]. Overall, individuals who have completed treatment for localized PCa must learn to manage these side effects alongside daily tasks such as maintaining a healthy lifestyle and relationships with spouses, returning to work, and managing finances.

Individuals often report difficulties adjusting to life after treatment and poor communication with providers, spouses, and other family members [5]. While cancer patients often have support from their providers and clinical staff, family, and friends during active treatment, these sources of support may diminish after patients move into the one year “re-entry” phase after completing treatment [6, 7]. The re-entry phase can be time of uncertainty as individuals are often resuming or alternating their previous roles (e.g., employee, spouse, father, friend) while also managing post-treatment functional and psychosocial side effects as well as interpersonal relationships, potential financial toxicity, and other practical concerns. Extant literature has identified medical and social determinants of preparedness for individuals in the re-entry phase after completion of cancer treatment, including marital status, age, overall physical health, social support, illness perception, and depressive symptoms [8, 9, 6, 7].

Guided by stress and coping theories [10, 11], Stanton and colleagues’ conceptual framework for post-treatment adjustment specifies contributors for supportive needs during re-entry [12]. Interpersonal/environmental context, individual context, and disease-related context are proposed to impact four domains of post-treatment adjustment through an individual’s appraisal and coping processes: (a) emotional functioning, (b) physical health, (c) interpersonal relationships, and (d) life perspective and practical concerns. Identifying the specific contributors of post-treatment adjustment will help providers and researchers identify and help individuals at risk of poor coping and adjustment [8]. However, current literature has focused on breast cancer [9, 6] or collapsed different cancer types into one group [8, 13], limiting the identification of specific factors associate with supportive needs among those that have completed treatment for localized PCa. Therefore, adapting Stanton and colleagues conceptual framework for post-treatment adjustment (Figure 1), this study aimed to identify medical and social determinants associated with self-efficacy for re-entry, perceived quality of interactions with medical providers, and practical concerns among individuals in their first year post-treatment for localized PCa.

## Methods

### Study design and participants.

A cross-sectional study was completed using baseline data from a randomized controlled trial evaluating a web-based support system for individuals within one year of treatment completion for localized PCa [14]. Individuals were eligible if they were (a) 18 years or older; (b) diagnosed with localized PCa; (c) within one year of treatment completion; (d) had access to a computer with Internet; (e) able to communicate in English; and (f) competent to give consent. Recruitment occurred between the years of 2013 and 2016 at four Mid-Atlantic cancer centers. Research and clinic staff identified eligible participants through medical chart review. Eligible participants were recruited during routine post-treatment clinic visits and research staff confirmed eligibility with interested participants. Enrolled participants provided written consent and completed the baseline assessment via their preferred method: online via REDCap, over the telephone, or via mail with a pre-addressed and stamped return envelope. Institutional Review Board approval was obtained at each study site. Participants received a $20 gift card after completing the baseline survey.

### Measures.

All study measures are validated with acceptable reliability and use health communication best practices. Demographic items included all variables available from the baseline survey and medical records: age, race/ethnicity, marital status, annual household income, and education. Medical variables included type of treatment completed [surgery, radiation (internal or external), or other (multiple treatment types or other treatment such as hormone therapy)], comorbidities [15], health literacy [16], and clinically significant depressive symptoms [17]. Clinically depressive symptoms was dichotomized based on clinical cutoff of 9 or higher.

#### Outcome variables.

*Self-efficacy for re-entry* is an author-constructed 14-item scale that measured participants’ self-efficacy to manage aspects of their physical (e.g., manage treatment related fatigue), interpersonal (e.g., maintain good relationships with friends), and mental health (e.g., manage stress, cope with fears about cancer recurrence) after completing treatment. Each item is assessed using an 11-point Likert-type scale from 0 (*not at all confident)* to 10 (*completely confident*). The scale also has four subscales: *social support* (4 items), *healthy lifestyle* (2 items), *treatment side effect coping* (5 items), and *emotional coping* (3 items). A mean score is calculated for the total scale and subscales with higher scores indicating greater self-efficacy. The scale and subscales have acceptable internal reliability (αs>.70).

Participants’ perceptions of their *medical interactions* (e.g., difficulty asking doctors questions, doctor’s don’t explain what they are doing to me) was assessed using a 5-item scale from the Cancer Rehabilitation Evaluation System [18]. Each item is assessed using a 5-point Likert-type scale from 0 (*not much*) to 4 (*very much*). A scale sum is calculated with higher scores indicating a poorer evaluation of their medical interactions. The scale had acceptable internal reliability (α=.76).

*Practical concerns* were assessed using an adapted 12-item scale that assessed participants’ concerns about managing the practical (i.e., tangible) aspects of their lives such as employment, diet and exercise, health insurance, and family responsibilities [19]. Each item is assessed using a 5-point Likert-type scale from 1 (*strongly disagree*) to 5 (*strongly agree*). A mean total score is calculated with higher scores indicating greater concerns. The scale demonstrated high internal reliability (α=.92).

### Statistical Analysis.

Univariate statistics were completed for all variables (i.e., frequencies, means). Bivariate analyses were completed to assess the relationship between demographic and medical variables (i.e., treatment completed, comorbidities, health literacy, depressive symptoms) with the outcome variables. Non-parametric tests (e.g., Mann–Whitney U, Kruskal–Wallis test) were used due to non-normality of the outcome variables. Variables were included in the multivariable regression models if they had a *p*-value of .10 or less with the outcome variable. Multivariable linear regression analyses were completed to identify factors associated with self-efficacy to re-entry, medical interactions, and practical concerns. Analyses were completed using IBM SPSS Statistics version 24.

## Results

A total of 431 participants were enrolled and completed the baseline survey. Participants had a mean age of 63.53 (SD=7.09; Range=42–86) and were predominantly Non-Hispanic White (72.8%) or Non-Hispanic Black (21.8%; Table 1). A majority of participants were married (80.7%) and approximately half had a household income over $75,000 (55.2%). Most participants had surgery (61.4%) or radiation (25.6%). Approximately one in four participants had clinically significant depressive symptoms (26.5%). Overall, participants reported high self-efficacy for re-entry (total score M=8.78, SD=1.11, max score=10) with responses ranging from 3 to 10. Most participants reported positive interactions with their medical providers, (M=2.55, SD=3.18, max score=20). Lastly, participants reported few practical concerns (M=1.76, SD=0.87, max score=5), however responses ranged from 0 to 5.

### Self-efficacy for re-entry.

Table 2 summarizes the multivariable linear regression analysis for the self-efficacy for re-entry total score and subscales (social support, healthy lifestyle, treatment side effect coping, emotional coping). Variables included in the models for the self-efficacy for re-entry total score and four subscales were age, race/ethnicity, marital status, income, education, comorbidities, health literacy, and clinically significant depressive symptoms (*ps*<.10 in bivariate analyses with self-efficacy for re-entry total score).

#### Self-efficacy for re-entry total score.

Non-Hispanic Black participants reported lower self-efficacy for re-entry compared to Non-Hispanic White participants (*β*=−.11, *p*<.05). Additionally, participants with greater income (*β*=.14, *p*<.05) and greater health literacy (*β*=.20, *p*<.001) had greater self-efficacy. Further, participants with greater number of comorbidities (*β*=−.09, *p*<.05) or having clinically significant depressive symptoms (*β*=−.40, *p*<.001) had significantly worse self-efficacy.

#### Self-efficacy for maintaining social support.

Non-Hispanic Black participants had lower self-efficacy for maintaining social support compared to Non-Hispanic White participants (*β*=−.11, *p*<.05), as did participants who had clinically significant depressive symptoms (*β*=−.31, *p*<.001). Participants with greater health literacy reported greater self-efficacy for maintaining social support (*β*=.19, *p*<.001).

#### Self-efficacy for maintaining a healthy lifestyle.

Participants with greater income (*β*=.13, *p*<.05) and health literacy (*β*=.20, *p*<.001) reported greater self-efficacy for maintaining a healthy lifestyle. However, participants with clinically significant depressive symptoms had lower self-efficacy for maintaining a healthy lifestyle (*β*=−.34, *p*<.001).

#### Self-efficacy for coping with treatment side effects.

Non-Hispanic Black participants reported significantly lower self-efficacy to cope with treatment side effects than Non-Hispanic White participants (*β*=−.12, *p*<.05). Similarly, participants with more comorbidities (*β*=−.16, *p*<.05) or who had clinically significant depressive symptoms (*β*=−.32, *p*<.001) had lower self-efficacy. Participants with greater income (*β*=.17, *p*<.05) or health literacy (*β*=.17, *p*<.05) reported greater self-efficacy for coping with treatment side effects.

#### Self-efficacy for emotional coping.

Participants who had clinically significant depressive symptoms reported lower self-efficacy for emotional coping (*β*=−.45, *p*<.001). However, participants with greater income (*β* =.12, *p*<.05) or health literacy (*β*=.16, *p*<.05), as well as older participants, had greater self-efficacy for emotional coping.

### Medical interactions.

Table 3 summarizes the multivariable linear regression analyses for medical interactions. Variables included in the model were race/ethnicity, income, education, health literacy, and clinically significant depressive symptoms (*ps*<.10 in bivariate analyses). Non-Hispanic Black participants (*β*=.14, *p*<.05) and participants of all other races (*β*=.13, *p*<.05) reported poorer interactions with their medical providers compared to Non-Hispanic White participants. Additionally, participants with clinically significant depressive symptoms had poorer interactions with medical providers (*β*= 27, *p* <.001). Conversely, participants with greater income (*β*=−.11, *p*<.05) or health literacy (*β*=−.18, *p*<.05) reported better interactions with their medical providers.

### Practical concerns.

Table 4 summarizes the multivariable linear regression analyses for practical concerns. Variables included in the model were age, race/ethnicity, income, education, type of treatment completed, health literacy, and clinically significant depressive symptoms (*ps*<.10 in bivariate analyses). Younger participants reported more practical concerns than older participants (*β*=−.23, *p*<.001). Additionally, participants of all other race/ethnicities [i.e., American Indian or Alaska Native (AI/AN), Asian, Hispanic] had more practical concerns than Non-Hispanic White participants (*β*=.12, *p*<.05). Participants with greater income (*β*=−.19, *p*<.001) or health literacy (*β*=−.11, *p*<.05) had fewer practical concerns. Participants who had surgery had more practical concerns than participants who had radiation (*β*=−.11, *p*<.05). Finally, participants who had clinically significant depressive symptoms had more practical concerns (*β*=.26, *p*<.001).

## Discussion

Several social and medical variables were significantly related to supportive needs for adapting to new roles among individuals that completed treatment for localized PCa. Notably, race/ethnicity, income, health literacy, and clinically significant depressive symptoms were significantly related to all three readiness domains (i.e., self-efficacy for re-entry, medical interactions, practical concerns). Additionally, age, comorbidities, and treatment completed were associated with some of the domains. These findings suggest certain localized PCa patients may be at greater risk for reduced coping ability, symptom management, and successfully returning to previous or adapted roles. This study is the next step towards identifying social and medical risk factors associated with supportive needs for individuals in their first year post-treatment for localized PCa and provides a foundation to future development and implementation of clinical support tools to help providers identify and support those at risk for continued poorer coping and management.

Our findings identified several disparities between Non-Hispanic White participants and participants of all other race/ethnicities. First, Non-Hispanic Black participants reported significantly less self-efficacy for re-entry (total score), as well as the social support and treatment side effects coping subscales, compared to Non-Hispanic White participants. Second, both Non-Hispanic Black participants and participants of all other race/ethnicities reported significantly worse interactions with their medical providers compared to Non-Hispanic White participants. Finally, participants of all other race/ethnicities (i.e., AI/AN, Asian, Hispanic) had more practical concerns compared to Non-Hispanic White participants. These findings suggest individuals who do not identify as Non-Hispanic White are experiencing greater difficulties navigating their medical care after treatment completion. While our study did not assess medical mistrust or provider implicit racial bias, these may be negative characteristics of the current health system associated with individuals’ perceived quality of care, patient-provider communication, and supportive needs. Non-Hispanic Black, AI/AN, and Hispanic patients have reported high rates medical mistrust with their medical providers [20–26]. Patients’ medical mistrust is often rooted in the patients’ belief that physicians did not respect them, discredited their symptoms, [20] spent an inadequate amount of time listening to the patients, and not sufficiently explaining treatment options [24]. Implicit bias is the “unconscious and involuntary attitudes which lie below the surface of consciousness, but can influence affect, behavior, and cognitive processes” and has been linked to patient medical mistrust and satisfaction with care [27]. Oncologists with higher levels of implicit racial bias have less patient-centered communication and shorter interactions with Non-Hispanic Black patients, negatively impacting patient confidence in provider-recommended treatments [28]. While best practices for antiracist training have not yet been identified, implicit racial bias is present as early as the first year of medical training suggesting training should begin as early as possible [27].

Health literacy has been linked to reduced physical, emotional, and functional well-being [29–31], poorer cancer care coordination [29, 32], and lower confidence in healthcare management [33]. Similarly, our study identified a positive association between health literacy and self-efficacy for re-entry and negative associations with quality of medical interactions and practical concerns. These findings suggest a need for provider training to improve communication with patients with the goal to meet supportive needs and improve adapting to roles in their daily lives during the re-entry period. As it is often difficult for providers to accurately assess patient health literacy, patient-provider communications training should focus on effective communication techniques across health literacy levels [34].

Financial toxicity—the financial burden faced by cancer patients—has been linked to overall poor quality of life, reduced quality of care, and greater mortality risk [35]. Individuals under financial hardship report financial distress, dissatisfaction with their medical care, as well as medical cost and wage concerns and are at risk of poor overall well-being and depression [36]. Our study identified associations between household income with supportive needs domains among individuals that have completed treatment for localized PCa. Participants with lower household income reported less self-efficacy for re-entry, including self-efficacy maintaining a healthy lifestyle and coping with treatment side effects and emotions; poorer interactions with their medical providers; and more practical concerns (e.g., job, family, and social responsibilities, health insurance). Our findings, along with the extant literature demonstrating the persistent harm of financial toxicity, illustrates the need for interventions such as supportive domestic help, financial assistance, expanding affordable care, and employment protection policies to help individuals manage financial costs after cancer treatment while maintaining overall quality of life [37].

Approximately 25% of participants in our study reported clinically significant depressive symptoms through the CES-D scale and is consistent with other studies [38, 39]. Study participants with clinically significant depressive symptoms had lower self-efficacy for re-entry, including all four subscales, poorer interactions with medical providers, and more practical concerns. As individuals experience clinical depression after PCa treatment completion at rates greater than the general population [40, 39], depression immediately post-treatment may exacerbate individuals’ ability to effectively manage treatment side effects and navigate practical concerns (e.g., employment, relationships). Although depressive symptoms often decrease during the first year post-treatment [41], depression has been linked to cancer-related and general health worry [42], as well as functional difficulties years after treatment completion [42, 38]. The American Cancer Society guidelines encourage depression screening and management for individuals after PCa treatment. However, research suggests certain populations are at risk for missed depression diagnoses (e.g., Black participants, unemployment, younger age, low income) [39] and one in four cancer patients may not be receiving adequate treatment for their depression [43]. Our findings suggest that not only should providers consistently screen for depression among all individuals who have completed treatment for PCa and provide adequate management or refer to other providers as needed, but also discuss post-treatment concerns—both medical and non-medical—among individuals with depression and provide appropriate resources and support.

Younger participants reported more practical concerns and lower self-efficacy for emotional coping than older participants in our study. This may due to greater perceived work and family obligations among younger individuals compared to those that are reaching or in retirement. In fact, financial toxicity is more commonly reported among younger individuals who have had cancer [44]. However, other studies have found older age is linked to reduced employment, early retirement, and longer sick leave [45]. Similarly, older individuals may differ in their perspectives and resources contributing to greater self-efficacy for emotional coping. Additional research may be warranted to better understand the unique perceptions between younger and older individuals and the specific concerns they have during this post-treatment phase.

Participants with greater comorbidities reported less self-efficacy for re-entry, including self-efficacy for managing treatment side effects. Research has linked comorbidities with cancer-related symptoms and worry [42], symptom management [8], reduced employment status [45, 46], depression [39], and reduced quality of life [47, 48] among individuals who have had cancer. Individuals treated for localized PCa with comorbidities may have greater concern about managing both their cancer-related symptoms alongside other illnesses. Medical providers should work together to build a supportive plan with their patients to help to increase confidence during re-entry and ultimately maintain quality of life.

Finally, our study identified greater practical concerns among participants that had surgery compared to those that had radiation. While urinary incontinence and sexual dysfunction is greater among individuals that have surgery, urinary irritation is often worse among those that had radiation [49]. Research has found that while individuals that have surgery for localized PCa treatment often report urinary incontinence, this does not impact work ability [50]. However, experiencing urinary incontinence may increase individuals’ perceptions about managing various aspects of their daily lives during the re-entry period. Providers should discuss patients’ concerns with managing treatment side effects and related concerns about managing a healthy lifestyle and other responsibilities (e.g., family, job).

As the number of individuals that have successfully treated localized PCa continues to grow, researchers and medical providers must address the difficult transition into routine daily roles after treatment completion. Our study identified several social and medical risk factors of supportive needs for re-entry illustrating gaps in current patient care that should be addressed. While cancer patients may have different experiences than individuals experiencing other chronic illness, our findings demonstrate some consistency with illnesses such as heart disease [51], chronic kidney disease [52, 53], irritable bowel syndrome [54], and endometriosis [55], suggesting commonalities across patient populations that may provide insight for future research. Our findings also suggest additional research is warranted to confirm these social and medical risk factors as well as effective, disseminable interventions that can be easily integrated into clinical care.

### Limitations.

Our number of enrolled participants that identified as American Indian/Native American, Asian, and Hispanic were too low to allow for race/ethnicity-specific analyses. This limits our ability to understand the specific psychosocial concerns among individuals of these race/ethnicities. Future research should ensure recruitment plans that will allow for sufficient participant recruitment of various race/ethnicities that are often understudied [56, 57]. Our study also did not assess sexuality or gender identity. Sexual and gender minorities often report poorer quality of life outcomes after treatment for PCa and other cancers [58, 59] and poorer satisfaction with medical care [60, 61], and extant literature has not adequately included sexual and gender minorities in research analyses, leaving a significant gap in our understanding of their needs [62]. Future research must assess sexual and gender identity during data collection to begin filling this critical knowledge gap. Additionally, our recruitment focused on localized PCa patients at four mid-Atlantic academic cancer centers—two National Cancer Institute (NCI)-Designated Comprehensive Cancer Centers, and one NCI-Designated Cancer Center, and one academic, non-NCI-Designated cancer center. The patient care and resources at these academic, predominately NCI-Designated cancer centers may differ from other oncology care localized PCa patients may receive and limits our ability to understand if our findings are specific to those completing treatment for localized PCa or if they persist across other patient populations. Our findings also may be limited in their generalizability outside of the U.S. as specific social factors may differ greatly (e.g., race/ethnicity and implicit bias, financial toxicity due to healthcare environment), while others may have commonalities (e.g., depressive symptoms, age, health literacy). Further, as participant enrollment was for a larger randomized controlled trial evaluating a web-based intervention, participant eligibility included access to a computer with Internet. This eligibility requirement limits our evaluation of psychosocial concerns to those with possibly greater access to health information and resources.

### Conclusions and implications.

This study identified several social and medical risk factors associated with supportive needs for adapting in the first year post-treatment for localized PCa. Specifically, this study found four risk factors associated with three supportive needs domains, highlighting a significant need for clinicians and researchers to evaluate and improve current patient-provider communication practices and support. Future research should further explore the perceptions of individuals who completed treatment for localized PCa longitudinally to identify changing supportive needs among this population over time, alongside the perspectives of clinicians, including oncologists and family medicine physicians, nurses, and other medical providers, to develop and implement practice guidelines to help individuals manage the medical and non-medical aspects of their daily lives.

## Figures and Tables

**Figure 1.**
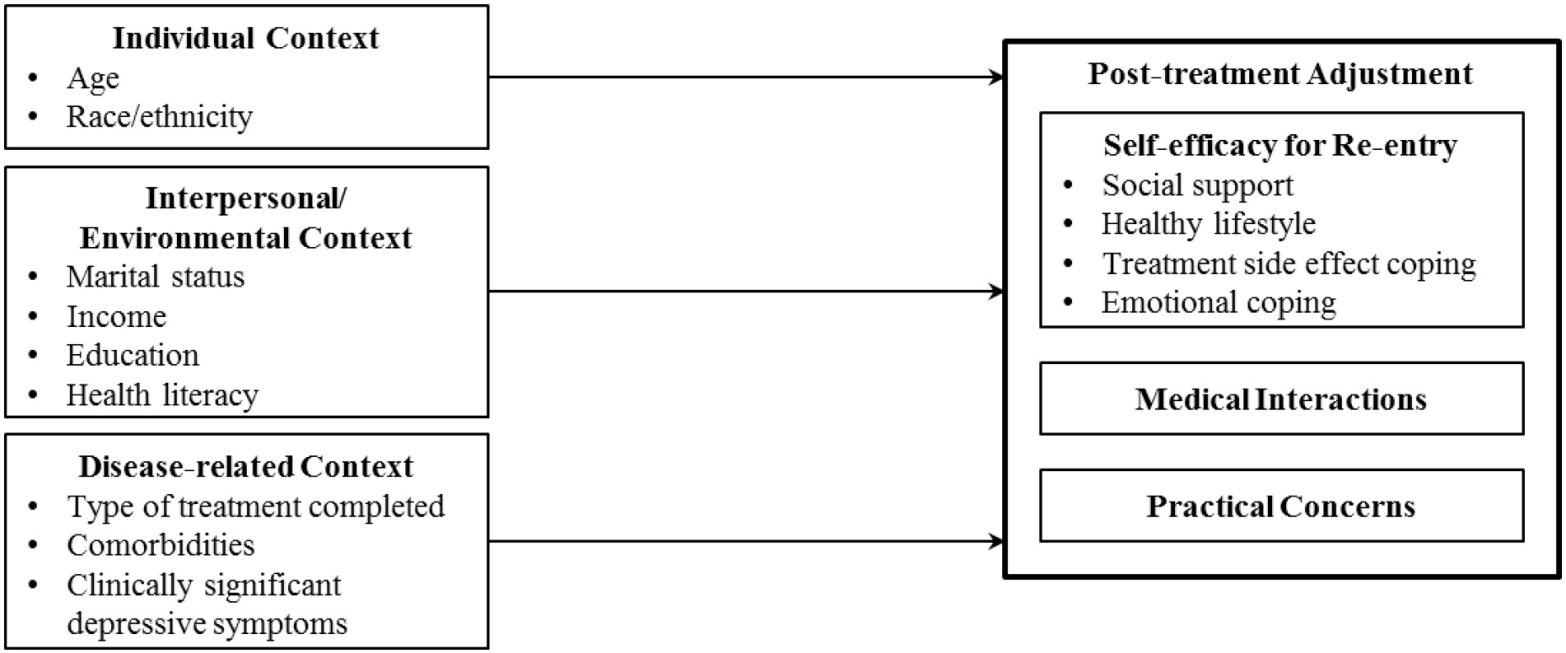
Adaptation of conceptual framework for post-tratment adjustment from Stanton et al., 2005

**Table 1.** Participant demographics (N=431)

Characteristic	n (%) or *M* (SD)
Age	63.53 (7.09)
Race/Ethnicity	
Non-Hispanic White	311 (72.8)
Non-Hispanic Black	93 (21.8)
All other races	23 (5.4)
Married	347 (80.7)
Household income	
$0 – 15,000	25 (6.2)
$15,001 – 30,000	24 (5.9)
$30,001 – 45,000	33 (8.1)
$45,001 – 60,000	49 (12.1)
$60,001 – 75,000	51 (12.6)
≥ $75,001	224 (55.2)
Education	
≤ High school diploma/GED	100 (23.4)
Some college/vocational school	127 (29.7)
Bachelor’s degree	101 (23.7)
Graduate degree	99 (23.2)
Treatment completed	
Surgery	261 (61.4)
Radiation	109 (25.6)
Other (e.g., hormone therapy, multiple treatments)	55 (12.9)
Charlson comorbidity index	0.38 (0.89)
Health literacy (max score: 15)	13.09 (2.33)
Clinically significant depressive symptoms	112 (26.5)
Self-efficacy for re-entry total Score (max score: 10)	8.78 (1.11)
Social support	9.33 (0.99)
Healthy lifestyle	9.09 (1.14)
Treatment side-effects	8.27 (1.50)
Emotional coping	8.71 (1.37)
Medical interactions (max score: 20)	2.55 (3.18)
Practical concerns (max score: 5)	1.76 (0.87)

**Table 2.** Multivariable regression analyses of self-efficacy for re-entry

	Overall score			Social support			Healthy lifestyle			Treatment side-effect coping			Emotional coping		
Variable	B	SE	*β*	B	SE	*β*	B	SE	*β*	B	SE	*β*	B	SE	*β*
Age	0.01	.01	.07	0.01	.01	.04	< 0.01	.01	< .01	0.01	.01	.07	0.02	.01	.10*
Race/ethnicity^a^															
Non-Hispanic Black	−0.32	.13	−.11*	−0.26	.13	−.11*	−0.21	.14	−.07	−0.46	.18	−.12*	−0.22	.16	−.06
All other races	−0.06	.21	−.01	−0.01	.21	< .01	0.34	.23	.07	−0.24	.29	−.04	−0.11	.26	−.02
Married^b^	0.01	.13	< .01	0.08	.13	.03	0.01	.15	< .01	−0.07	.18	−.02	0.05	.16	.01
Income	0.10	.04	.14*	0.01	.04	.02	0.09	.04	.13*	0.16	.05	.17*	0.11	.05	.12*
Education	−0.05	.03	−.07	−0.04	.03	−.07	−0.06	.04	−.09	−0.04	.05	−.04	−0.06	.04	−.07
Comorbidities	−0.11	.06	−.09*	0.01	.06	.01	−0.03	.06	−.02	−0.27	.08	−.16*	−0.06	.07	−.04
Health literacy	0.10	.02	.20**	0.08	.02	.19**	0.10	.03	.20**	0.11	.03	.17*	0.10	.03	.16*
Depressive symptoms	−1.02	.11	−.40**	−0.70	.11	−.31**	−0.89	.12	.34*	−1.10	.16	.32**	−1.42	.14	−.45**
R^2^			.35			.18			.23			.31			.34
F			21.98**			9.06**			12.69**			18.59**			21.34**

* *p* < .05;

** *p* < .001

a Reference group: Non-Hispanic White

b Reference group: Single/divorced/widowed/separated

**Table 3.** Multivariable regression analysis of medical interactions

Variable	B	SE	*β*
Race/ethnicity^a^			
Non-Hispanic Black	1.09	.37	.14*
All other races	1.66	.61	.13*
Income	−0.22	.11	−.11*
Education	0.01	.10	< .01
Health literacy	−0.23	.07	−.18*
Depressive symptoms	1.82	.31	.27**
_R_2			.22
F			17.76**

* *p* < .05;

** *p* < .001

a Reference group: Non-Hispanic White

**Table 4 .** Multivariable regression analysis of practical concerns

Variable	B	SE	*β*
Age	−0.03	.01	−.23**
Race/ethnicity^a^			
Non-Hispanic Black	−0.12	.10	−.05
All other races	0.43	.17	.12*
Income	−0.10	.03	−.19**
Education	−0.04	.03	−.07
Type of treatment completed^b^			
Radiation	−0.22	.09	−.11*
Other	< 0.01	.12	< .01
Health literacy	−0.04	.02	−.11*
Depression	0.50	.09	.26**
R^2^			.28
F			15.94**

* *p* < .05;

** *p* < .001

a Reference group: Non-Hispanic White

b Reference group: Surgery
